# Right pneumothorax during endoscopic balloon dilation for oesophageal stricture following tracheoesophageal fistula surgery: a case report

**DOI:** 10.3389/fped.2026.1825565

**Published:** 2026-05-12

**Authors:** Heya Yu, Yuangui Lin

**Affiliations:** 1 Department of Anesthesiology, West China Second University Hospital, Sichuan University, Chengdu, China; 2 Key Laboratory of Birth Defects and Related Diseases of Women and Children (Sichuan University), Ministry of Education, Chengdu, China

**Keywords:** cardiopulmonary resuscitation, case report, endoscopic balloon dilation, oesophageal stricture, right pneumothorax

## Abstract

Perioperative pneumothorax, when accompanied by decreased heart rate and oxygen saturation, constitutes a critical intraoperative emergency and poses a significant emergency management challenge to the anaesthetist. We report a case of a two-month-old girl who underwent a third endoscopic balloon dilation for oesophageal stricture following surgery for a tracheoesophageal fistula. During balloon dilation, a significant and persistent decrease in heart rate and oxygen saturation occurred. Following cessation of the procedure, we initiated cardiopulmonary resuscitation and administered intravenous medications. Heart rate and oxygen saturation briefly improved then again subsequently declined. After ruling out excessive depth of the endotracheal tube, vagal reactions and airway spasm, a right-sided pneumothorax was identified in the patient. Prompt closed-thoracic drainage was performed, after which the patient's heart rate and oxygen saturation returned to normal. The patient developed right pneumothorax during the fourth balloon dilation. This case highlights that balloon dilation for oesophageal stricture may cause oesophageal rupture and oesophageal fistula, leading to pneumothorax. Prompt diagnosis and thoracostomy were crucial.

## Introduction

Congenital tracheoesophageal fistula (TEF) is a severe and rare condition in the neonatal period that can be life-threatening. Congenital TEF is often detected following birth when infants present with symptoms such as choking, cyanosis, frothy sputum, and feeding difficulties (1). Ladd's classification indicates five Congenital TEF subtypes: Type I represents isolated oesophageal atresia; Type II involves oesophageal atresia with proximal TEF; Type III involves oesophageal atresia with a distal TEF, which is the most prevalent and accounts for 80–90% of cases. Type III is further subdivided into Type IIIa and IIIb, which is dependent upon whether the distance between the two blind ends exceeds or falls below 2 cm. Type IV TEF presents without oesophageal atresia, also termed H-type fistula. Type V TEF involves oesophageal atresia with both proximal and distal TEF. This condition generally requires surgical intervention. Common complications following TEF repair, such as oesophageal stricture, gastroesophageal reflux disease, and dysphagia, significantly impact the child's quality of life. Oesophageal stricture, in particular, necessitates treatment with repeated hospital admissions (2, 3). Balloon dilation is a widely accepted technique for addressing oesophageal strictures (4). We report a case of right-sided pneumothorax complicated by decreased heart rate and oxygen saturation during endoscopic balloon dilation for oesophageal stricture, necessitating emergency resuscitation. This case report was conducted with the written consent of the patient's parents and conformed to Case Report guidelines (5).

## Case description

**Figure 1:**
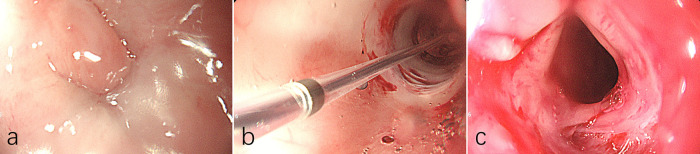
Endoscopic balloon dilation for oesophageal stricture. **(a)** Before balloon dilation, granulation tissue obstructing the oesophageal lumen. **(b)** Balloon dilation in progress, the balloon was inflated to a maximum pressure of 5 atmospheres with an outer diameter of 11 mm. **(c)** After balloon dilation, examination of the dilated oesophageal segment showed no evidence of perforation internally.

A 2-month-old female infant presented with choking, cyanosis, and frothy sputum after birth. Examination revealed a type IIIb TEF. She underwent TEF repair surgery 6 days after birth. One month after the surgery, the patient experienced choking and vomiting episodes. Further examination revealed oesophageal strictures. The gastroenterology department had previously performed two oesophageal balloon dilation procedures, both of which were successful. This was therefore the third oesophageal balloon dilation treatment. The paediatric patient underwent general anaesthesia with endotracheal intubation. A standard endoscope (outer diameter approximately 8.9 mm) was introduced orally. At a distance of approximately 10 cm from the incisors, an oesophageal stricture measuring approximately 4 mm in diameter was observed, with granulation tissue obstructing the oesophageal lumen (Figure 1a). A single-use balloon dilator was deployed, and the stricture ring was dilated twice. The balloon was inflated to a maximum pressure of 5 atmospheres (ATM) with an outer diameter of 11 mm (Figure 1b). Endoscopic examination revealed dilation of the oesophagus, narrowing with partial mucosal laceration. During dilation, the patient experienced transient decreases in heart rate and oxygen saturation, which resolved following completion of the procedure.

**Figure 2:**
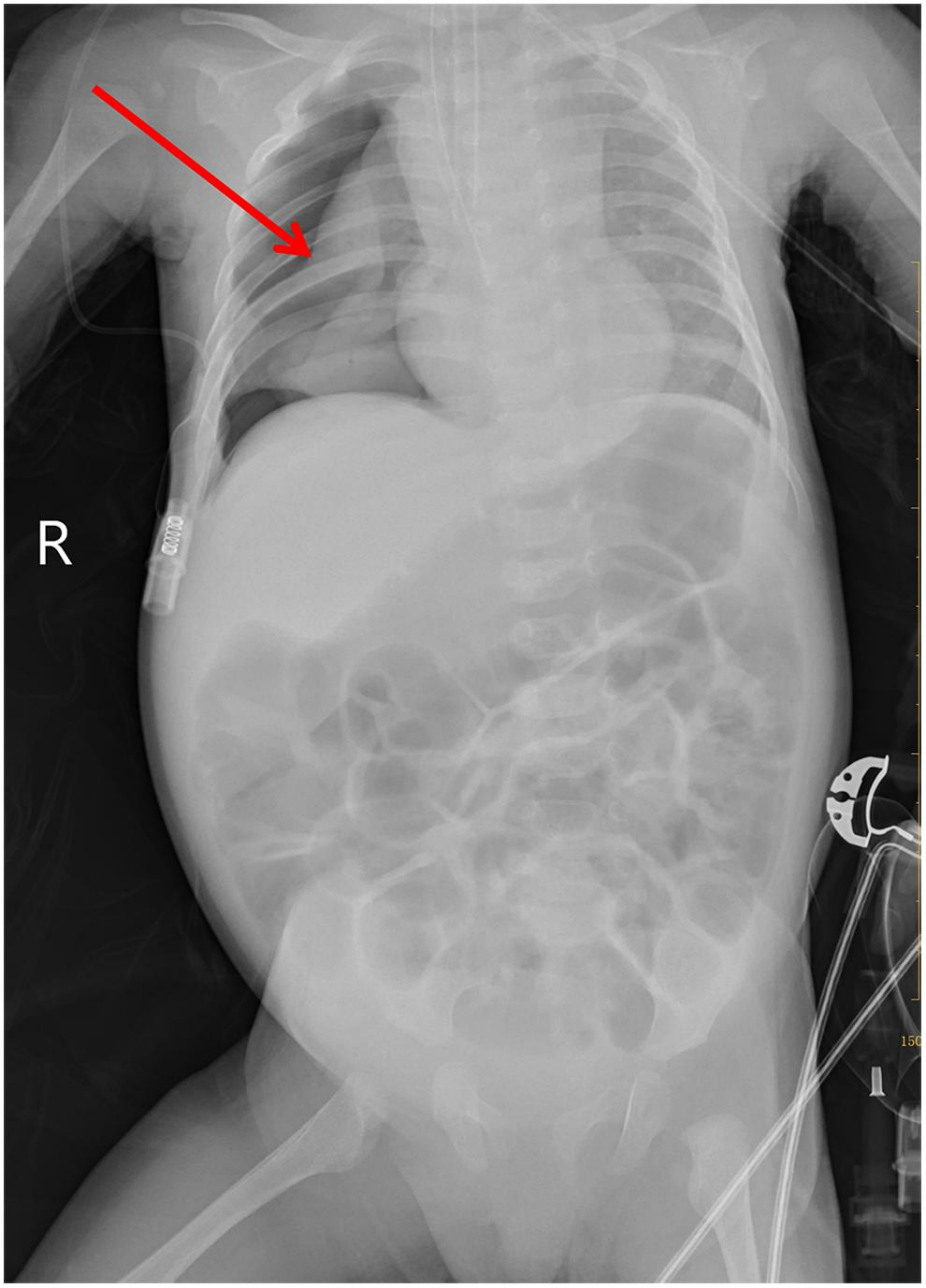
Right-sided pneumothorax. Pneumothorax compressed about 60% of the lung tissue; widespread air in the abdominal intestines, with no signs of pneumoperitoneum; patient intubated.

**Figure 3:**
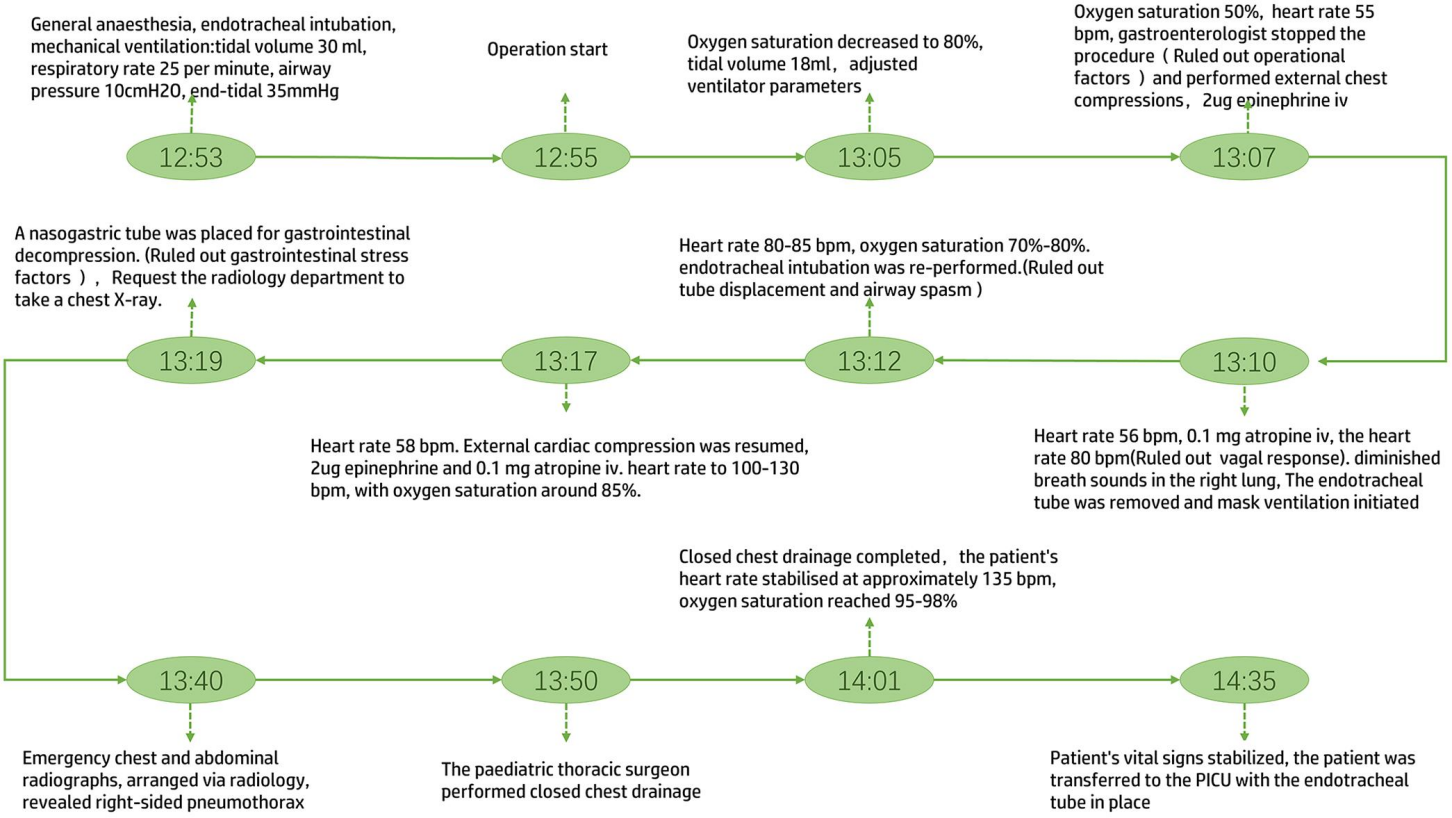
Timeline of events.

Following dilation, resistance was encountered during passage of the standard gastroscope through the stricture. The gastroenterologist proceeded with a third dilation, applying a maximum balloon pressure of 5 ATM. with an external balloon diameter of 11 mm. At 13:05, during the dilation, the patient's oxygen saturation gradually decreased to 80%. The anaesthetist adjusted ventilator parameters, but the oxygen saturation continued to fall to 50%, with heart rate decreasing to 55 beats per minute (bpm) and blood pressure at 72/41 mmHg. The gastroenterologist was immediately summoned to cease the procedure. External cardiac compression was initiated and 2 μg of epinephrine administered. At 13:10, the patient's heart rate remained at 56 bpm. Following intravenous administration of 0.1 mg atropine, the heart rate increased to 80 bpm, blood pressure rose to 82/47 mmHg, but with oxygen saturation at 60%. Auscultation revealed diminished breath sounds in the right lung. The endotracheal tube was removed and mask ventilation initiated. The patient's heart rate fluctuated between 80 and 85 bpm, with oxygen saturation fluctuating between 70%–80%. At 13:12, endotracheal intubation was re-performed. The patient's heart rate fluctuated between 80 and 90 bpm, with oxygen saturation around 80%. At 13:17, the patient's heart rate again decreased to 58 bpm. External cardiac compression was resumed, and intravenous administration of 2 μg epinephrine and 0.1 mg atropine was administered. The patient's heart rate subsequently increased to 100–130 bpm, with oxygen saturation around 85%. Blood pressure was 83/46 mmHg. Auscultation revealed persistently diminished breath sounds in the right lung. A nasogastric tube was placed under endoscopic guidance for gastrointestinal decompression. Examination of the dilated oesophageal segment showed no evidence of perforation internally (Figure 1c). Emergency chest and abdominal radiographs, arranged via radiology, revealed right-sided pneumothorax (Figure 2). Following a physical examination and review of the chest X-ray, the paediatric thoracic surgeon diagnosed a pneumothorax and immediately performed closed chest drainage on the right side. After draping and disinfecting the area, select the second intercostal space on the right mid-clavicular line as the puncture site. Insert the needle vertically along the upper margin of the rib; after aspirating to confirm the position, insert the drainage tube to a depth of 8 cm. Bubbles were observed after connecting to the water-sealed bottle. Subsequently, the patient's heart rate stabilised at approximately 135 bpm, oxygen saturation reached 95%–98%, and blood pressure was 85/46 mmHg. Following confirmation of stable vital signs, the patient was transferred to the Paediatric Intensive Care Unit (PICU) with the endotracheal tube in place. We have created a timeline illustrating the entire sequence of events (Figure 3).

**Figure 4:**
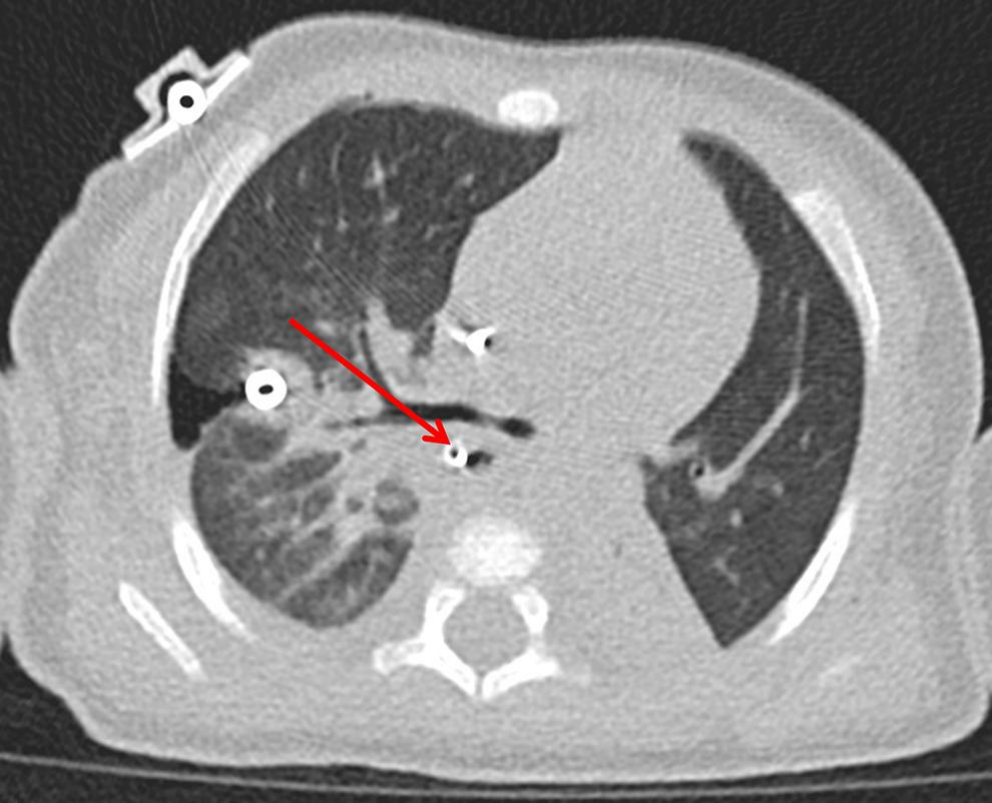
High-resolution CT. 1. A tube is visible in the right chest wall and pleural cavity; small right-sided pneumothorax with approximately 20% compression of the right lung. 2. A tubular shadow is visible within the oesophagus, an air-filled balloon-like shadow protruding from the right side of the middle segment of the oesophagus outside the oesophageal contour. 3. Bilateral pneumonia; partial consolidation and atelectasis in the lower lobe of the left lung.

After the patient's condition stabilized, a high-resolution CT scan revealed an air-filled balloon-like shadow protruding from the right side of the middle segment of the esophagus outside the esophageal contour (Figure 4). Considering the patient's condition, it was highly likely that there was an esophageal rupture and esophageal fistula. However, the fistula had the potential for self-repair. Therefore, closed thoracic drainage was performed to observe the patient's pneumothorax condition. On the 6th day after the surgery, the patient's tracheal tube was removed, and a chest X-ray showed that the pneumothorax was less than 5%. Subsequently, the patient's vital signs remained stable, breathing was normal, and on the 10th day after the surgery, the patient was transferred back to the general ward. The closed thoracic drainage tube was removed on the 11th day after the operation.

On the 21st day post-surgery, the gastroenterologist considered performing a fourth esophageal dilation for the patient due to esophageal stricture. After thorough preoperative preparation, tracheal intubation under general anesthesia was performed. However, during the dilation procedure, the patient experienced a drop in oxygen saturation to 88% and a decrease in heart rate to 86 bpm. The gastroenterologist immediately halted the procedure, and the anesthesiologist administered 1 µg/kg of epinephrine. Although the heart rate recovered, the oxygen saturation did not improve significantly. Upon auscultation, breath sounds in the right lower lung were diminished, and a high suspicion of pneumothorax was raised based on ultrasound examination. We immediately inserted an 18-gauge blunt-tipped needle into the second intercostal space at the midline of the right clavicle; oxygen saturation was seen to recover to 93% almost immediately, after which we called for a thoracic surgeon to attend. A thoracic surgeon was promptly consulted to perform a closed chest drainage. Once the patient's vital signs stabilized, the patient was transferred to the PICU with the endotracheal tube in place.

## Discussion

Perioperative sudden pneumothorax is a critical and serious condition. Failure to detect and address it promptly may endanger the patient's life. Given its sudden onset and urgency, it poses a significant challenge for anesthesiologists. The literature reports that tracheal and bronchoscopic procedures carry a higher risk of pneumothorax (6, 7).There have been case reports of cardiac arrest caused by perioperative pneumothorax requiring ECOM support therapy (8). The primary treatment for pneumothorax is urgent thoracic decompression (9). Wu et al. reported a case of tension pneumothorax caused by the accidental entry of a nasogastric tube into the right pleural cavity during balloon dilation for oesophageal stricture, which required thoracotomy for oesophageal repair (10). In balloon dilation procedures for esophageal strictures, cases of suspected pneumothorax caused by esophageal perforation and fistula formation are extremely rare. Our case serves as a cautionary reminder.

Endoscopic balloon dilation is the current preferred treatment for oesophageal strictures. Oesophageal perforation due to endoscopic dilation is considered one of the rare adverse events, with a rate of 0.53–0.6% per intervention (11). Common risk factors currently reported in association with oesophageal perforation caused by dilation include chemical or malignant lesions, as well as a history of previous surgery (12). Both at our centre and in the literature, it is recommended that the procedure be performed under general anaesthesia with endotracheal intubation (13). Clinical practice indicates that balloon dilation exerts pressure on the airway and heart, leading to transient decreases in heart rate and oxygen saturation. This is one of the reasons why pneumothorax was initially difficult to promptly detect. In our case, both the first and second balloon dilations resulted in transient decreases in heart rate and oxygen saturation. These conditions could be restored after the dilations were completed. However, after the third dilation was stopped, there was a persistent, unresolved decrease in heart rate and oxygen saturation. After removing the tracheal tube and performing mask ventilation, we reinserted the tracheal tube and thoroughly ruled out whether the tracheal tube was too deep or airway spasm occurrence. Lung auscultation and chest x-ray examination helped us promptly detect and manage pneumothorax, thereby stabilizing the child's critical condition. When the second pneumothorax occurred, we dealt with it more promptly and conducted an immediate ultrasound diagnosis. Ultrasound, when readily available, is also recommended for diagnosing pneumothorax, with greater speed and accuracy (14).

The patient developed pneumothorax during the third and fourth balloon dilations, while the first two procedures were successful using the same pressure. Upon review, the chest radiograph taken on the third day post-first surgery revealed a small pneumothorax on the right side, indicating the presence of a minor fistula. Subsequently, the fistula site healed spontaneously. These two balloon dilation may have coincidentally targeted the weakened area, thereby causing a recurrence of pneumothorax. van Stigt et al. also reported instances of re-rupture at the oesophageal fistula site (15).

## Conclusion

This case report documents that balloon dilation for oesophageal stricture may cause oesophageal rupture and oesophageal fistula, leading to pneumothorax. Prompt diagnosis and early placement of closed-thoracic drainage to resolve pneumothorax are crucial for the child's resuscitation.

## Data Availability

The original contributions presented in the study are included in the article/Supplementary Material, further inquiries can be directed to the corresponding author.
